# Audio-guided self-hypnosis for reduction of claustrophobia during MR imaging: results of an observational 2-group study

**DOI:** 10.1007/s00330-021-07887-w

**Published:** 2021-04-15

**Authors:** Adriane E. Napp, Torsten Diekhoff, Olf Stoiber, Judith Enders, Gerd Diederichs, Peter Martus, Marc Dewey

**Affiliations:** 1 Department of Radiology, Charité - Universitätsmedizin Berlin Campus Mitte, Humboldt-Universität zu Berlin, Freie Universität Berlin, Charité Platz 1, 10117 Berlin, Germany; 2 Hypnovita, Academy of Hypnotic Medical Arts, Josephsburgstraße 33, 81673 Munich, Germany; 3 Department of Radiology, Charité - Universitätsmedizin Berlin Campus Vircho-Klinikum, Humboldt-Universität zu Berlin, Freie Universität Berlin, Augustenburger Platz 1, 13353 Berlin, Germany; 4 Clinical Epidemiology and Applied Biometry, Universitätsklinikum Tübingen, Silcherstraße 5, 72076 Tübingen, Germany

**Keywords:** Phobic disorders, Anxiety, Magnetic resonance imaging, Hypnosis

## Abstract

**Objectives:**

To evaluate the influence of audio-guided self-hypnosis on claustrophobia in a high-risk cohort undergoing magnetic resonance (MR) imaging.

**Methods:**

In this prospective observational 2-group study, 55 patients (69% female, mean age 53.6 ± 13.9) used self-hypnosis directly before imaging. Claustrophobia included premature termination, sedation, and coping actions. The claustrophobia questionnaire (CLQ) was completed before self-hypnosis and after MR imaging. Results were compared to a control cohort of 89 patients examined on the same open MR scanner using logistic regression for multivariate analysis. Furthermore, patients were asked about their preferences for future imaging.

**Results:**

There was significantly fewer claustrophobia in the self-hypnosis group (16%; 9/55), compared with the control group (43%; 38/89; odds ratio .14; *p* = .001). Self-hypnosis patients also needed less sedation (2% vs 16%; 1/55 vs 14/89; odds ratio .1; *p* = .008) and non-sedation coping actions (13% vs 28%; 7/55 vs 25/89; odds ratio .3; *p* = .02). Self-hypnosis did not influence the CLQ results measured before and after MR imaging (*p* = .79). Self-hypnosis reduced the frequency of claustrophobia in the subgroup of patients above an established CLQ cut-off of .33 from 47% (37/78) to 18% (9/49; *p* = .002). In the subgroup below the CLQ cut-off of 0.33, there were no significant differences (0% vs 9%, 0/6 vs 1/11; *p* = 1.0). Most patients (67%; 35/52) preferred self-hypnosis for future MR examinations.

**Conclusions:**

Self-hypnosis reduced claustrophobia in high-risk patients undergoing imaging in an open MR scanner and might reduce the need for sedation and non-sedation coping actions.

**Key Points:**

• *Forty percent of the patients at high risk for claustrophobia may also experience a claustrophobic event in an open MR scanner.*

• *Self-hypnosis while listening to an audio in the waiting room before the examination may reduce claustrophobic events in over 50% of patients with high risk for claustrophobia.*

• *Self-hypnosis may also reduce the need for sedation and other time-consuming non-sedation coping actions and is preferred by high-risk patients for future examinations.*

**Supplementary Information:**

The online version contains supplementary material available at 10.1007/s00330-021-07887-w.

## Introduction

Up to 10% of patients cannot undergo magnetic resonance (MR) imaging due to severe claustrophobia triggered by the small diameter of the MR tube or loud noises [1, 2]. In view of recent studies indicating that, with appropriate precautions, pacemakers and implantable cardioverter-defibrillators (ICD) no longer preclude MR examinations [3, 4], severe claustrophobia is the main remaining relative contraindication to MR imaging. Patients may experience anxiety when encountering MR imaging or being inside the MR tube during the examination [5], especially if the procedure is unknown to them and they lack information about what to expect [6]. Claustrophobic events are common but their prevalence varies with patients’ socioeconomic background and medical history [7]. Dealing with claustrophobic patients is a challenge for medical staff [8, 9]. Besides the feeling of confinement, the noise of the machine during the scan has been identified as a major factor inducing fear and discomfort [2, 10]. However, there are many factors that influence claustrophobia in general and during MR imaging in particular [11].

Distress during acquisition may lead to premature termination of the scan or reduce image quality due to motion artifacts, impeding proper interpretation of images [6]. Therefore, many patients need conscious sedation to complete the examination [12, 13], which requires monitoring of vital functions and exposes patients to the risk of severe complications [14]. Furthermore, medical sedation requires an experienced operator to ensure maximum efficiency, which is not available in all hospitals or outpatient centers [15]. To avoid medications or to at least reduce their dose and hence the risks, several alternative strategies have been proposed and investigated to reduce patients’ fear during MR imaging [16–18]. Hypnosis and relaxation techniques have been found to reduce patients’ anxiety and the need for medication during medical procedures [19–22]. Hypnosis may be considered as a modified state of consciousness that emphasizes attention, concentration, and the letting go of thoughts and is characterized by mental relaxation, altered perception of the environment, and disengagement of the discursive and critical analytical reasoning [23]. While many hypnotherapists stress that hypnotic suggestions have to be internalized before being effective, the definition of self-hypnosis remains vague. For this manuscript, the hypnotic intervention was defined as self-hypnosis because a sufficient amount of effort originated from the patient and no hypnotherapist was present. From an economic point of view, strategies that do not prolong the examination or preparation are of particular interest here as MR imaging is already expensive compared to other imaging modalities. Therefore, relaxation techniques or so-called self-hypnosis may be an interesting and effective strategy to reduce the need for oral or intravenous sedation, while at the same time keeping the patient comfortable through the examination.

We hypothesize that audio-guided self-hypnosis before MR imaging can reduce claustrophobic events in patients at an increased risk of claustrophobia during MR imaging in an open scanner. Therefore, the aim of our study was to evaluate self-hypnosis in patients with an increased risk of anxiety and claustrophobia compared to a control group of patients who also presented with this increased risk but were examined on the same MR scanner without self-hypnosis. The focus of the analysis was on the frequency of incomplete examinations, use of medical sedation, and non-sedation coping strategies. We furthermore assessed patients’ satisfaction with and acceptance of self-hypnosis as preparation for their examination.

## Materials and methods

### Ethics approval and informed consent

The study was approved by the local institutional review board (EA1/237/12). All patients in the intervention group gave written informed consent. No written informed consent was required and waived by the institutional review board from patients in the control cohort, who were only asked to complete the German version of the validated English-language claustrophobia questionnaire (CLQ) before the MR examination [24]. During recruitment, we noted a difference in gender distribution compared with the control cohort. Therefore, an amendment to the original ethics approval was granted to investigate more male patients.

### Study design

The prospective intervention cohort included patients who were scheduled to undergo an MR examination on our 1.0-T open MR scanner (Panorama, Philips Healthcare). These were mostly patients with known claustrophobia or patients with discomfort during previous examinations for whom the referring physicians or the patients themselves asked for an examination on an open MR system. Thus, we expected a high-risk cohort. All other inclusion and exclusion criteria were the same as for the control group [11]. Exclusion criteria were referrals from the intensive care unit, severe emergencies, general poor health, severe psychological disorders, invasive procedures during MR imaging (e.g., biopsies), age under 18, and examinations specifically scheduled for other studies. Furthermore, patients were excluded if they were not able to answer the questions of the claustrophobia questionnaire (CLQ) prior to the examination. Patients were included in the analysis if they remained in the MR scanner for at least 20 min, even if their examinations could not be completed because of technical problems. These examinations were not counted as premature terminations due to claustrophobia. If patients underwent two examinations with self-hypnosis, only the first examination and the corresponding questionnaire data were used for this analysis.

At a time during the study, patients in both cohorts (intervention and control) were offered and granted medical sedation or coping actions to make them feel confident to try self-hypnosis. This approach enabled an intention-to-treat analysis.

### Self-hypnosis intervention

A hypnotherapist (OS) informed of the specific needs of patients in the MR scanner created a self-hypnosis script which included self-empowering and metaphoric suggestions addressing the worries of patients inside an MR scanner and designed to reduce claustrophobic sensations during the examination (Appendix 1). While the script utilizes elements of several different hypnotic styles, it is mostly characterized by an indirect phrasing, much in the fashion of a modified so-called Ericksonian hypnosis. It was recorded as a 23-min audio file with background music and provided to the patient with an easy-to-use MP3 player and high-quality earphones (Bose AE2) while waiting and preparing for the examination. Patients and technicians could stop self-hypnosis at any time and had to stop for beginning the examination. During longer waiting periods, patients could restart the audio file. The exposure time to the self-hypnosis script was measured for each patient. There was no audio stimulation, especially no self-hypnosis, during the examination itself. Technicians were advised to handle the high-risk patients in the same way as their other patients.

### Study conduct

All patients willing to participate completed the CLQ (Appendix 2) before the hypnotic intervention and after the MR examination. The CLQ consists of 26 questions picturing the patient in different situations and asking to which extent these induce fear — ranging from 0 (no fear) to 4 (maximum fear) [24]. A CLQ mean score was calculated by dividing the sum of all scores by the number of questions. Only questionnaires where all 26 questions were answered were considered. We recorded specific events during the examination that led to premature termination or a coping action and the reason why this event took place (e.g., claustrophobia or pain). An examination was also counted as prematurely terminated, if acquired images were non-diagnostic due to motion artifacts. Intravenous and oral sedation (using benzodiazepine) was used according to the guidelines of the American Society of Anesthesiology [25]. Non-sedation coping actions included a trial run with the patient in the scanner, a pause, an accompanying person in the scanner room, mirror glasses, or a specific, comforting conversation addressing issues of claustrophobia in the scanner. All events were protocoled, and it was explicitly assessed if the coping action was due to claustrophobia or possible other causes such as pain.

Besides completing the CLQ for a second time, patients were also asked to answer some questions after the examination describing their experience during the examination and self-hypnosis. The questions related to previous experiences with MR imaging and claustrophobic events, their subjective assessment of the usefulness of self-hypnosis, and if they would prefer further examinations with or without self-hypnosis (satisfaction questionnaire, Table 1).

**Table 1:** Selected items and possible answers and number of answers of the satisfaction questionnaire. The satisfaction questionnaire was answered by 53 patients after magnetic resonance imaging (*n*, number of patients). Not all patients completed all questions. Questions 1 and 1a do not refer to the study MR examination but to previous experiences.

No	Item of the satisfaction questionnaire	Answers				
1	Did you ever experience claustrophobia before, during, or after an MR examination previously?	No	Yes	Several times		
		(*n* = 17)	(*n* = 28)	(*n* = 8)		
1a	Did you need support before or during previous examinations?	No	Sedative injection	Oral sedation tablet	Prone position	
		(*n* = 10)	(*n* = 11)	(*n* = 5)	(*n* = 0)	
		Test run	Pause	Prism glasses	Escort in scanner room	
		(*n* = 2)	(*n* = 1)	(*n* = 4)	(*n* = 1)	
2	How useful did you find self-hypnosis for this examination?	Not at all	A little	Moderately	Useful	Very useful
		(*n* = 12)	(*n* = 3)	(*n* = 14)	(*n* = 13)	(*n* = 11)
3a	Which kind of examination would you prefer for future examinations on the open MR scanner?	MR with self-hypnosis	MR without self-hypnosis		I don’t know	
		(*n* = 35)	(*n* = 11)		(*n* = 6)	
3b	Which kind of examination would you prefer for future examinations on a conventional MR scanner with a normal bore?	MR with self-hypnosis	MR without self-hypnosis		I don’t know	
		(*n* = 29)	(*n* = 5)		(*n* = 11)	

### Definition of a claustrophobic event

The occurrence of a claustrophobic event was assumed if the scan terminated prematurely or if claustrophobia required intravenous or oral sedation or a non-sedation coping action at any time before or during the examination to complete the scan. Claustrophobic events were ranked by severity, ranging from high (premature termination/non-diagnostic image quality), to moderate (intravenous or oral sedation), to low (non-sedation coping action). On the patient level, the most severe event counted for analysis — e.g., if a patient needed oral sedation and caused motion artifacts precluding diagnosis, the examination counted as prematurely terminated.

### Control group

For comparison, we used the data from a cohort of patients with a similar risk of claustrophobia who were examined on the same 1.0-T open MR scanner. These patients also completed the CLQ before the examination but did not receive self-hypnosis. The study design has already been reported. However, the data of this specific cohort was not included by Napp et al in the analysis [11].

### Statistical analysis

The statistical sample size was estimated by an expert statistician based on the hypothesis of a reduction of claustrophobic events from 43% (38 of 89) to 20% (9 of 46) by self-hypnosis. A two-tailed type 1 error of .05, a power of more than 80%, and an expected drop-out rate of 10% yielded a total sample size of 51 patients. We performed a Mann-Whitney *U*-test to compare the self-hypnosis and control groups regarding age and CLQ score and a chi-square test for gender distribution. In logistic regression analyses with outcomes “claustrophobic events in total” and the subcategories “premature termination,” “sedation,” and “non-sedating coping actions,” the effect of self-hypnosis was adjusted for age, gender, and CLQ. We performed a Wilcoxon matched-pairs signed-rank test to test for significant differences in CLQ mean values before and after self-hypnosis and the MR examination. We also compared the occurrence of events in patients with a CLQ below an established cut-off (.33 for all patients, Appendix 3) [11] and those above the cut-off using Fischer’s exact test (below cut-off) and a chi-square test with Yates’ correction (above cut-off). Furthermore, events in both groups were analyzed by type of examination. Statistical tests were executed using Prism (Version 6.0 for Mac, GraphPad Software Inc.) and SPSS (Version 23.0, IBMCorp). A *p* value equal to or smaller than .05 was considered significant.

## Results

**Fig. 1:**
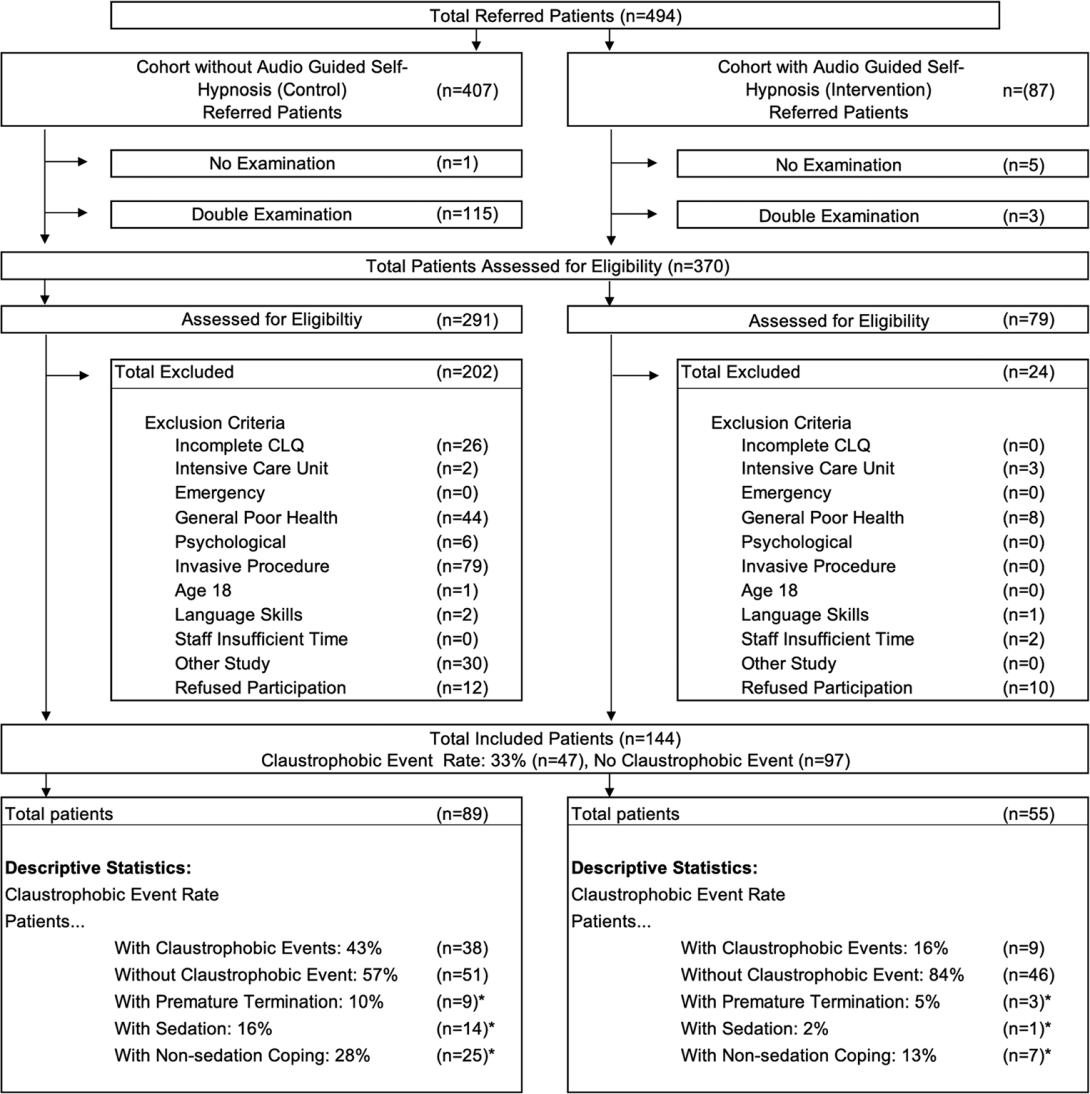
Flow chart of study inclusion in the non-hypnosis and the self-hypnosis cohorts. The cohort without self-hypnosis is a retrospectively evaluated patient population examined on the same MR scanner. In both cohorts, only the first examination was included. A total of 124 (25%) of 494 referred patients were excluded because they did not undergo an examination for different reasons or were scheduled twice. In the control cohort, a majority of patients were excluded due to invasive procedures or general poor health. In the self-hypnosis cohort, 10 (12%) of 87 patients were excluded because they refused to participate. Overall, 144 (29%) of 494 patients were included in the analysis. Given is the number of all noted claustrophobic events. *Further analysis only includes the most severe event per patient

During the study period, 87 examinations were conducted on the 1.0-T open MR scanner. Of these examinations, we included 58 examinations of patients willing to participate in the study, answered the CLQ prior to the examination, and used self-hypnosis for a mean of 23.3 min (20 to 40 min). Three patients participated twice and, therefore, the last examination was excluded. Among the patients with repeated examinations, there was a 52-year-old woman with a relatively high CLQ (mean 2.1) who completed the first examination without need for sedation or non-sedation coping actions using self-hypnosis. However, for the second examination, she asked for additional oral sedation. Another patient was not offered self-hypnosis for the second examination because of time constraints. Despite being able to complete the first MR examination without any coping actions besides the interventional self-hypnosis, she needed intravenous sedation for the second scan. None of the patients were included in both the control and the intervention groups. Figure 1 shows a flow chart of study inclusion. Both groups had a mean CLQ value above the established cut-off of .33 [11]. The descriptive statistics of the intervention and the control group can be found in Table 2 and detailed information of anatomic regions in Table 3.

**Table 2:** Demography and predictors of self-hypnosis (intervention) in comparison to the control group. CLQ, claustrophobia questionnaire. Data are given in percentage (nominator/denominator) or mean ± standard deviation and (min–max)

	Intervention (*n* = 55)		Controls (*n* = 89)	
Sex				
Male	31%	(17/55)	51%	(45/89)
Female	69%	(38/55)	49%	(44/89)
Age	53.6 ± 13.9	[25–81]	51.2 ± 14.3	[19–82]
CLQ	1.71 ± 1.0	[0–3.8]	1.51 ± 1.0	[0–4.0]
Claustrophobic events (total)	16%	(9/55)	43%	(38/89)
Premature termination for claustrophobia	5%	(3/55)	10%	(9/89)
Sedation for claustrophobia	2%	(1/55)	16%	(14/89)
Coping for claustrophobia without sedation	13%	(7/55)	28%	(25/89)

**Table 3:** Number of exams from different anatomical regions and corresponding claustrophobic event rate in both cohorts

Examination	Intervention group (*n* = 55)				Control group (*n* = 89)			
	Examinations		Events		Examinations		Events	
Combinations	1	(2%)	0	(–)	12	(13%)	6	(50%)
Brain/head/neck	15	(27%)	4	(27%)	35	(39%)	17	(46%)
Thorax	4	(7%)	1	(25%)	3	(3%)	1	(33%)
Abdomen/pelvis	24	(44%)	4	(17%)	27	(31%)	11	(41%)
Upper extremities	5	(9%)	–	(0/5)	5	(6%)	3	(60%)
Lower extremities	6	(11%)	–	(0/6)	7	(8%)	0	(-)

**Fig. 2:**
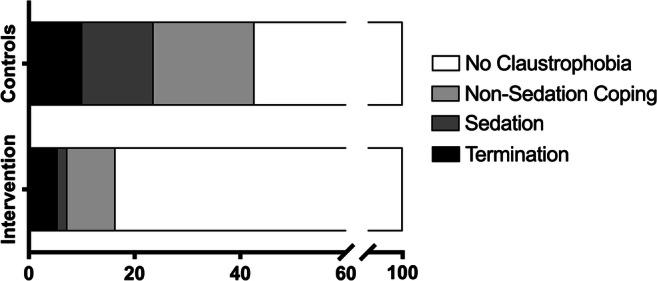
Frequency of claustrophobia (percentage of patients) in intervention and control groups. Overall, there were fewer claustrophobic events, non-sedation and sedation coping actions, and premature terminations in the self-hypnosis group compared to the control group

Use of audio-guided self-hypnosis reduced claustrophobic events from 43% (38/89) to 16% (9/55), the need for sedation from 16 to 2% (14/89 vs 1/55), and non-sedation coping actions from 28 to 13% (25/89 vs 7/55, Table 2 and Fig. 2). Although the frequency of premature terminations was halved (3/55 vs 9/89), the difference was not statistically significant. The results of the multivariate logistic regression analysis are shown in Table 4 (univariate results are shown in Appendix 4). Whereas events in patients above the CLQ cut-off of .33 decreased significantly (9% vs 47%; 1/11 vs 37/78; *p* = .002), no significant decrease was seen in patients below the cut-off (0% vs 9%; 0/6 vs 1/11; *p* = 1.0).

**Table 4:** Multivariate analysis for claustrophobic events in total and event subgroups. There were significantly less events in total, need for sedation and non-sedation coping actions in the intervention group compared to the controls after adjustment for gender, age and CLQ-values

	Claustrophobic Events (total)		Premature Termination		Sedation for Claustrophobia		Non-Sedation Coping	
	(47 events)		(12 events)		(15 events)		(32 events)	
	Odds ratio	*p* -value	Odds ratio	*p* -value	Odds ratio	*p* -value	Odds ratio	*p* -value
Sex^1^	---	.24	---	.58^5^	---	.90	---	.81
Age^2^	---	.44	---	.89	---	.71	---	.40
CLQ^3^	**3.54**	**< .001**	---	.069	---	.067	**2.40**	**< .001**
Intervention^4^	**.14**	**< .001**	---	.33	**.099**	**.028**	**.28**	**.012**

^1^Male vs. female

^2^1 year

^3^1 point

^4^Yes vs. no

^5^*p* values for inclusion in forward variable selection procedure with p_in = .05, p_out = .10.

In the intervention cohort, 11% of patients (6 of 55) terminated the MR examination prematurely, 5% (3/55) due to claustrophobia, 4% (2/55) due to a technical problem with the MR scanner, and 2% (1/55) due to a reduced general condition and pain. In the control group, there were 12% (11/89) premature terminations, 10% (9/89) due to claustrophobia, and 2% (2/89) for technical reasons.

There was no intravenous sedation in the intervention cohort (0/55) compared to 7% (6/89) in the control group (*p* = .06). Oral sedation was required by 4% of the patients using self-hypnosis (2/55) — among them one who demanded sedation for pain and not for claustrophobia — compared to 9% (8/89) in the control group (*p* = .5).

Regarding non-sedation coping actions for claustrophobia, we observed extended conversations in 5% (3 of 55) of the self-hypnosis group compared to 26% (23 of 89) in the control group (*p* = .003), 4% (2/55) test runs (controls 12% (11/89), *p* = .13), 4% (2/55) pauses (controls 3% (3/89), *p* = > .99), 2% (1 of 55) escort person (controls 3% (3/89), *p* = .66), and no (0%) other, unspecified coping action (controls 8%; 7/89).

All patients completed all CLQ questions before the examination. Fifty patients answered all CLQ questions after the examination. There was no statistically significant difference in CLQ scores before and after the examination (1.6 ± .92 vs 1.62 ± 1.1; *p* = .79). All 53 patients who answered the satisfaction questionnaire had a previous MR examination, none of them in an open MR. The mean usefulness rating of self-hypnosis assigned by the patients was 2.23 ± 1.35, with scores ranging from 0 (not at all helpful) to 4 (very helpful) and 2 being neutral. However, 67% (35/52) of the patients preferred self-hypnosis on an open MR scanner. For examinations on a conventional MR imager, self-hypnosis was preferred by 64% (29/45) of the patients. This question was not answered by all patients, in some cases because patients would not undergo an examination on a conventional MR scanner at all. More details can be found in Table 1.

## Discussion

To our knowledge, this is the first study addressing the influence of self-hypnosis on claustrophobic events in patients undergoing MR examinations. The main findings of our study are (*a*) audio-guided self-hypnosis significantly reduced claustrophobic events from 43 to 16% in high-risk patients; (*b*) the need for conscious sedation decreased from 16 to 2%; and (*c*) the need for time-consuming coping actions was reduced from 28 to 13% by audio-guided self-hypnosis. Self-hypnosis and the experience with MR imaging did not result in higher CLQ scores directly after imaging, and most patients (67%; 35/52) stated that they would prefer future MR imaging with support of self-hypnosis.

### Clinical workflow

The approach of audio-guided self-hypnosis presented here is easy to integrate into clinical workflow and saves time for the medical staff compared to individual hypnosis by an expert therapist. Self-hypnosis presented by means of an audio device can be handed over to the patient along with a short explanation when registering in the radiology department or provided on a smartphone application beforehand. The patient can use the file at his or her discretion while waiting for the examination and completing the information sheet and informed consent or use it for preparation and training at home. Our results show that self-hypnosis reduces the need for conscious sedation and non-sedation coping actions, which require extra staff and room time, medication and further patient care items as described by Bluemke et al [26]. In addition, self-hypnosis eliminates drug-related adverse events that may occur in some patients [26] and could thus be of special interest for outpatient centers, as self-hypnosis has no known negative effect on the patient’s ability to drive a car after the examination.

### Effect of hypnosis

Previous investigations show a positive effect of hypnosis on other medical procedures especially in terms of the need for medical sedation [19, 27–29]. Hypnosis can reduce the amount of medications needed or even completely replace benzodiazepines [22, 30–32]. Particularly, patients with comorbidities precluding sedating drug therapy or children might benefit from hypnosis [33, 34]. Self-relaxation techniques can be used by most patients referred for radiological procedures — complex scoring or assessment of hypnotic susceptibility is not required [35]. Hypnosis has thus been shown to reduce the cost of medical treatment during invasive radiologic procedures by Lang et al [36].

Some patients are still deterred by myths and misinformation about hypnosis and hypnotical trance, believing that they might lose control or experience an unwilling influence on their behavior [37]. However, we also face a growing number of patients showing a critical attitude toward drug therapy during information talks and therefore willing to use alternative treatments, which they believe to be less harmful. Therefore, self-hypnosis should be offered to patients to increase comfort and reduce the need for other coping strategies during MR.

### Limitations

The type of hypnosis might not fit all patients and could lose effect compared to individual hypnosis performed by a specialist. Furthermore, we were not able to assess the entering or deepness of the hypnotic state. Nevertheless, our results show that this type of self-hypnosis has beneficial effects. A selection bias may be present as our criteria favored inclusion of patients with a positive attitude, while excluding patients with a critical attitude to hypnosis and relaxation exercises. This bias might also lead to the observed higher proportion of women in the interventional cohort. Critical patients may be more difficult to hypnotize; however, most hypnotherapists stress that “believing” in hypnosis is not mandatory for hypnotic susceptibility [38]. Unfortunately, we have not enough information to present a detailed analysis of those patients, who refused to participate. Another limitation is that we did not randomize patients but used instead a historical control cohort. Therefore, there may be effects on our results that we are not aware of, e.g., due to the slightly different distribution of exam types in both cohorts. However, we compared results for gender, age, and the CLQ scores and corrected the statistics for confounders. We did not compare self-hypnosis such as offering relaxing music or a text without suggestions. Hence, attentive listening alone might have had an effect in individual patients. Furthermore, effects on image quality were not assessed for this analysis. Finally, the audio file that we designed has a total duration of 23 min. This might be slightly too long for daily practice; however, this can be adjusted. Furthermore, it was done by the patient alone without increased efforts by the staff.

## Conclusions

Audio-guided self-hypnosis reduced claustrophobia in patients undergoing imaging in an open MR scanner with a high risk based on the claustrophobia questionnaire. Self-hypnosis appears to be a valuable tool for reducing medical sedation and the time medical staff spends on coping strategies. Further studies are warranted to address the effectiveness of self-hypnosis compared to standard care and investigate its effect in a large, randomized, and sham-controlled trial.

## Supplementary Information

ESM 1 (DOCX 47 kb)

## Abbreviations

AUROC: Area under the receiver operation characteristic curve
CLQ: Claustrophobia questionnaire
ICD: Implantable cardioverter-defibrillators
LR: Likelihood ratio
MR: Magnetic resonance
NPV: Negative predictive value
PPV: Positive predictive value

## Declarations

### Guarantor

The scientific guarantor of this publication is Dr. rer. medic. Adriane Napp.

### Statistics and biometry

Prof. Dr. Peter Martus kindly provided statistical advice for this manuscript.

### Methodology

• prospective study with comparison to retrospective cohort

• performed at one institution
