# Supplementary material for: Audio-guided self-hypnosis for reduction of claustrophobia during MR imaging: results of an observational 2-group study
Source: Eur Radiol. 2021 Apr 15;31(7):4483–91. doi: 10.1007/s00330-021-07887-w (PMC8213599; doi:10.1007/s00330-021-07887-w)
Supplement: Supplementary file 1 — (DOCX 47 kb) [file 330_2021_7887_MOESM1_ESM.docx]

**ELECTRONIC SUPPLEMENTARY MATERIAL**

**Appendix 1: Flow Chart of the Self-Hypnosis Audio File with Design and Strategy.**

The script is divided into four parts (introduction, hypnotic induction, hypnotic intervention and hypnotic exduction). Basic elements of each part are listed. The self-hypnotic script was conceived and written following modern concepts that deliberately avoid giving any direct suggestions and utilize modern, indirect hypnotic language patterning, much in the fashion of the so-called Ericksonian hypnosis.

**Appendix 2: The Claustrophobia Questionnaire.**

|  | Not at all anxious (0) | | | Slightly anxious (1) | | | Moderately anxious (2) | | | Very anxious (3) | | | Extremely anxious (4) | | |
| --- | --- | --- | --- | --- | --- | --- | --- | --- | --- | --- | --- | --- | --- | --- | --- |
|  | I | A | C | I | A | C | I | A | C | I | A | C | I | A | C |
| 1. Locked in a small DARK room without windows for 15 minutes | 6 | 9 | 16 | 10 | 3 | 14 | 10 | 11 | 17 | 18 | 13 | 18 | 11 | 14 | 24 |
| 2. Locked in a small WELL LIT room without windows for 15 minutes | 18 | 14 | 33 | 9 | 10 | 11 | 15 | 12 | 24 | 10 | 12 | 11 | 3 | 3 | 10 |
| 3. Handcuffed for 15 minutes | 22 | 19 | 31 | 6 | 5 | 17 | 14 | 7 | 13 | 7 | 14 | 13 | 6 | 6 | 15 |
| 4. Tied up with hands behind back for 15 minutes | 19 | 16 | 23 | 6 | 7 | 16 | 11 | 4 | 12 | 7 | 12 | 19 | 12 | 12 | 19 |
| 5. Caught in tight clothing and unable to remove it | 17 | 16 | 16 | 7 | 8 | 18 | 8 | 8 | 15 | 13 | 9 | 18 | 10 | 10 | 22 |
| 6. Standing for 15 minutes in a straitjacket | 8 | 10 | 14 | 2 | 4 | 5 | 6 | 6 | 11 | 13 | 5 | 18 | 26 | 26 | 41 |
| 7. Lying in a tight sleeping bag enclosing legs and arms, tied at the neck, unable to get out for 15 | 11 | 13 | 14 | 5 | 4 | 9 | 6 | 7 | 9 | 8 | 4 | 19 | 25 | 23 | 38 |
| 8. Head first into a zipped up sleeping bag able to leave whenever you wish | 17 | 17 | 24 | 6 | 4 | 9 | 4 | 4 | 18 | 6 | 7 | 6 | 22 | 19 | 32 |
| 9. Lying in the trunk of a car with air flowing through freely for 15 minutes | 7 | 8 | 15 | 6 | 4 | 11 | 6 | 6 | 8 | 5 | 4 | 23 | 31 | 28 | 32 |
| 10. Having your legs tied to an immovable chair | 13 | 18 | 19 | 19 | 7 | 20 | 18 | 9 | 29 | 9 | 12 | 9 | 5 | 5 | 12 |
| 11. In a public washroom and the lock jams | 13 | 15 | 23 | 8 | 10 | 20 | 14 | 8 | 22 | 10 | 15 | 10 | 10 | 3 | 14 |
| 12. In a crowded train which stops between stations | 18 | 19 | 33 | 9 | 7 | 20 | 10 | 9 | 14 | 9 | 9 | 10 | 9 | 7 | 12 |
| 13. Swimming while wearing a nose plug | 24 | 24 | 44 | 6 | 8 | 19 | 10 | 6 | 13 | 8 | 9 | 11 | 7 | 4 | 2 |
| 14. Working under a sink for 15 minutes | 32 | 29 | 56 | 7 | 6 | 15 | 9 | 6 | 7 | 5 | 7 | 7 | 1 | 3 | 4 |
| 15. Standing in an elevator on the ground floor with the doors closed | 16 | 18 | 29 | 10 | 6 | 18 | 11 | 14 | 11 | 10 | 7 | 14 | 8 | 6 | 17 |
| 16. Trying to catch your breath during vigorous exercise | 37 | 30 | 59 | 5 | 6 | 9 | 7 | 9 | 10 | 4 | 2 | 6 | 2 | 4 | 5 |
| 17. Having a bad cold and finding it difficult to breathe through your nose | 28 | 28 | 46 | 7 | 6 | 23 | 11 | 10 | 10 | 6 | 6 | 5 | 3 | 1 | 5 |
| 18. Snorkeling in a safe practice tank for 15 minutes | 21 | 20 | 52 | 6 | 9 | 14 | 11 | 6 | 8 | 7 | 8 | 6 | 9 | 8 | 9 |
| 19. Using an oxygen mask | 22 | 23 | 42 | 7 | 6 | 21 | 8 | 6 | 13 | 8 | 9 | 10 | 10 | 7 | 3 |
| 20. Lying on a bottom bunk bed | 36 | 33 | 65 | 3 | 4 | 9 | 7 | 6 | 11 | 6 | 7 | 3 | 3 | 1 | 1 |
| 21. Standing in the middle of the 3^rd^ row at a packed concert realizing that you will be unable to leave until the end | 15 | 20 | 36 | 7 | 4 | 17 | 17 | 12 | 7 | 7 | 12 | 11 | 9 | 3 | 18 |
| 22. In the center of a full row at a cinema | 28 | 29 | 53 | 6 | 5 | 10 | 11 | 11 | 12 | 6 | 5 | 8 | 4 | 1 | 6 |
| 23. Working under a car for 15 minutes | 15 | 14 | 47 | 4 | 6 | 12 | 10 | 7 | 10 | 9 | 9 | 10 | 17 | 14 | 10 |
| 24. At the furthest point from an exit on a tour of an underground mine shaft | 11 | 11 | 27 | 9 | 8 | 14 | 5 | 5 | 11 | 13 | 11 | 17 | 17 | 15 | 20 |
| 25. Lying in a sauna for 15 minutes | 27 | 26 | 53 | 8 | 8 | 13 | 7 | 6 | 8 | 4 | 1 | 5 | 9 | 9 | 10 |
| 26. Waiting for 15 minutes in a plane on the ground with the doors closed | 28 | 0 | 46 | 9 | 2 | 16 | 8 | 13 | 7 | 4 | 1 | 12 | 6 | 4 | 8 |

Questions and results from the claustrophobia questionnaire: Absolute number of answers from the intervention cohort before (I; n=55) and after MRI and self-hypnosis (A; n=51/50) and from the control cohort (C; n=89). After the MRI, some patients did not answer all questions.

**Appendix 3 - Cutoff, AUC analysis, and parameters of diagnostic validity of the CLQ for all patients with 90% sensitivity.**

|  | **Mean** | **95% CI** | **Nominator/Denominator** |
| --- | --- | --- | --- |
| **Cut-off** | .33 |  |  |
| **AUROC** | .795 | .769-.822 |  |
| **Sensitivity** | .900 | .862-.929 | 279/310 |
| **Specificity** | .410 | .393-.427 | 1301/3174 |
| **PPV** | .130 | .116-.145 | 279/2152 |
| **NPV** | .977 | .967-.984 | 1301/1332 |
| **pos LR** | 1.525 | 1.455-1.599 |  |
| **neg LR** | .244 | .174-.342 |  |

Auroc = area under the ROC curve, PPV = positive predictive value, NPV = negative predictive value, pos LR = positive likelihood ration, neg LR = negative likelihood ration.

**Appendix 4 – Number of exams from different anatomical regions and corresponding claustrophobic event rate in both cohorts.**

| Examination | Intervention (n=55) | | | | Controls (n=89) | |  |  |
| --- | --- | --- | --- | --- | --- | --- | --- | --- |
|  | Exams | | Events | | Exams | | Events | |
| Combinations | 2% | (1/55) | - | (0/1) | 13% | (12/89) | 50% | (6/12) |
| Brain/Head/Neck | 27% | (15/55) | 27% | (4/15) | 39% | (35/89) | 46% | (17/35) |
| Thorax | 7% | (4/55) | 25% | (1/4) | 3% | (3/89) | 33% | (1/3) |
| Abdomen/pelvis | 44% | (24/55) | 17% | (4/24) | 31% | (27/89) | 41% | (11/27) |
| Upper Extremities | 9% | (5/55) | - | (0/5) | 6% | (5/89) | 60% | (3/5) |
| Lower Extremities | 11% | (6/55) | - | (0/6) | 8% | (7/89) | - | (0/7) |

Appendix 5 – Univariate analysis of claustrophobic events and subgroups depending on gender, age, CLQ-value and intervention.

|  | **Claustrophobic events (total)** | | **Premature Termination for Claustrophobia** | | **Sedation for Claustrophobia** | | **Coping for Claustrophobia without sedation** | |
| --- | --- | --- | --- | --- | --- | --- | --- | --- |
|  | **no** | **yes** | **no** | **yes** | **no** | **yes** | **no** | **yes** |
| **Sex**  **(female) ^1^** | 55/97  (56.7%) | 28/47  (59.6%) | 77/132  (58.3%) | 6/12  (50.0%) | 75/129  (58.1%) | 8/15  (53.3%) | 63/112  (56.3%) | 20/32  (62.5%) |
|  | *P* = .74 | | *P* = .58 | | *P* = .72 | | *P* = .53 | |
| **Age^2^** | 52.6  ± 14.7 | 51.2  ± 12.8 | 52.1  ± 14.4 | 52.7  ± 10.0 | 52.1  ± 14.5 | 52.6  ± 10.0 | 52.6  ± 14.0 | 50.5  ± 14.3 |
|  | *P* = .57 | | *P* = .887 | | *P* = .89 | | *P* = .47 | |
| **CLQ^2^** | **1.31**  **± .88** | **2.15**  **± .94** | 1.54  ± .99 | 2.07  ± .74 | 1.54  ± .98 | 1.95  ± .94 | **1.43**  **± .94** | **2.13**  **± .93** |
|  | ***P* < .001** | | *P* = .07 | | *P* = .13 | | ***P* < .001** | |
| **Intervention (yes)^1^** | **46/97**  **(46.6%)** | **9/47**  **(19.1%)** | 52/132  (39.4%) | 3/12  (25.0%) | **54/129**  **(41.9%)** | **1/15**  **(6.7%)** | **48/112**  **(42.9%)** | **7/32**  **(21.9%)** |
|  | ***P* = .001** | | *P* = .33 | | ***P* = .008** | | ***P* = .03** | |

^1^ number of cases (%), ^2^ mean ± standard deviation. There were significantly less events in total, need for sedation and non-sedation coping actions in the intervention group compared to the historical controls. The CLQ was significantly higher in patients with events and with need for non-sedation coping actions.
